# Milk protein-based formulas containing different oils affect fatty acids balance in term infants: A randomized blinded crossover clinical trial

**DOI:** 10.1186/s12944-017-0457-y

**Published:** 2017-04-14

**Authors:** Carolina Oliveira de Souza, Maria Efigênia Q. Leite, John Lasekan, Geraldine Baggs, Lorena Silva Pinho, Janice Izabel Druzian, Tereza Cristina M. Ribeiro, Ângela P. Mattos, José A. Menezes-Filho, Hugo Costa-Ribeiro

**Affiliations:** 1 Postgraduate Programme in Medicine and Health, Federal University of Bahia, Augusto Viana, s/n, Canela, Salvador, Bahia 40110-060 Brazil; 2 Fima Lifshitz Research Center at CHUPES, Federal University of Bahia, Augusto Viana, s/n, Canela, Salvador, Bahia 40110-060 Brazil; 3 Pediatric Nutrition R&D, Abbott Nutrition, Abbott Laboratories, Cleveland Avenue, Columbus, OH 43215-1724 USA; 4 College of Pharmacy, Federal University of Bahia, Postgraduate Programme in Food Science, Barão de Jeremoabo, s/n, Ondina, Salvador, Bahia 40170-110 Brazil

**Keywords:** Palm olein, Kernel palm oil, Fatty acid balance, Infant formula, Brazilian infants

## Abstract

**Background:**

Palm olein is used in infant formula fat blends in order to match the fatty acid profile of human milk. While the effects on fatty acid balance have been evaluated, the use of palm olein in combination with palm kernel oil and supplementation with docosahexaenoic acid (DHA) and arachidonic acid (ARA) has not been similarly assessed in infants. This study evaluated the effects of infant formulas containing different fat compositions on the balance of fat, fatty acids, and calcium.

**Methods:**

In this randomized, crossover, double-blinded study, 33 healthy term infants (68–159 ± 3 days of age at enrollment) were fed two formulas for 14 days in a tolerance period, followed by a 4-day metabolic balance period in 17 of the male subjects. The study compared two commercially available milk-based powdered formulas in Brazil; the PALM formula contained palm olein (44%), kernel palm oil (21.7%), and canola oil (18.5%) as the predominant fats, whereas the NoPALM formula contained other fat sources.

**Results:**

Fat absorption (%) was greater for NoPALM versus PALM-fed infants (96.55 and 95.50%, respectively; *p* = 0.023). The absorption percentage of palmitic acid (C16:0) did not differ significantly between formulas (*p* > 0.05), but this acid was excreted at significantly higher concentrations in the PALM (29.42 mg/kg/day) than in the NoPALM (12.28 mg/kg/day) formula groups. DHA and ARA absorption percentages were also higher in NoPALM-fed infants. Calcium absorption was higher in NoPALM-fed infants (58.00%) compared to those fed PALM (40.90%), but the difference was not significant (*p* = 0.104) when calcium intake was used as a covariate. However, calcium retention was higher in NoPALM-fed infants compared to that in PALM-fed infants with or without calcium intake as a covariate. Adverse events did not differ between groups (*p* > 0.05).

**Conclusions:**

The absorption of essential fatty acids was similar for both formulas; however, long-chain polyunsaturated fatty acids (DHA and ARA) were better absorbed from the NoPALM formula. Fat absorption and calcium retention were lower in term infants fed the PALM-based formula.

**Clinical trial registration:**

Clinicaltrial.gov # NCT00941564.

## Background

During the neonatal period, infants have a high demand for essential nutrients to provide adequate energy supply. In human milk as well as most infant formulas, 50% of the dietary calories are supplied to the newborn as fat, and more than 98% of milk fat is in the form of triglycerides, which contain saturated, monounsaturated, and polyunsaturated fatty acids of varying chain lengths esterified to glycerol [1]. On an energy basis, infant formulas should provide indispensable fatty acids in amounts at least equal to the reference fat (i.e., breast milk), irrespective of source [1–3].

The blends of vegetable oils used in infant formulas are selected to match the excellent absorption by the infant of breast milk fat, but aside from the absence of long-chain polyunsaturated fatty acids (LCPUFA), they differ considerably from human milk fat in their fatty acid profiles. Palmitic acid is the major saturated fatty acid in breast milk, accounting for 17–25% of the total [4, 5]. Palm oil and its low melting fraction, palm olein, a relatively inexpensive source of palmitic acid, are added to many infant formulas in amounts that mimic the palmitic acid content of human milk [6]. However, the positional distribution of individual fatty acids on the triacylglyceride molecules, which affects fat absorption, differs between these vegetable oils and human milk fat [7].

Most fatty acids are better absorbed as monoglycerides than as free acids because monoglycerides form mixed micellae with bile acids and cannot form complexes with divalent cations. Fatty acids in the sn-2 position are absorbed as soluble 2-monoacylglycerides, while those in the sn-1 and sn-3 positions are absorbed as free fatty acids [5]. The absorption of free fatty acids varies by structure: monounsaturated (90% of oleic acid (18:1n9) in breast milk, is in the sn-1 or −3 position), polyunsaturated, and saturated fatty acids containing 12 or fewer carbons are all well absorbed. Conversely, sn-2 palmitic monoglyceride is better absorbed than unesterified palmitic acid [5, 8].

In contrast to human milk fat, in which palmitic acid is esterified predominantly in the sn-2 position, palmitic acid is esterified predominantly in the sn-1 and sn-3 positions in vegetable oils added to infant formulas [5, 7]. In the sn-2 position, palmitic acid is generally not hydrolyzed by pancreatic lipase, and the remaining 2-monoacylglycerol is well absorbed [8]. However, palmitic acid in the sn-1 and sn-3 positions is hydrolyzed by pancreatic lipase; the produced free palmitic acid may form calcium–fatty acid complexes, resulting in reduced fat and calcium absorption [6, 8]. These complexes, known as calcium soaps, are insoluble, indigestible, and positively related to stool hardness. The formation of calcium soaps may partly explain the substantial differences in the absorption of nutrients (fat and calcium) and bowel habits between breast- and formula-fed infants [9, 10].

Significant clinical evidence has shown decreased absorption of fatty acids, fat, and calcium by infants fed formulas containing palm olein as the major fat compared to absorption in formulations without palm olein [4, 6, 9, 11, 12]. However, these studies only evaluated palm olein combined with other fats (soy, coconut, high oleic safflower, or sunflower oils) [4, 11–13]. Previously published data from same participant population as the current study demonstrated that calcium retention was lower in infants fed formulas containing palm olein associated with palm kernel/canola oils and supplemented with LCPUFA (docosahexaenoic acid - DHA and arachidonic acid - ARA) [9]. However, that study did not evaluate the individual balance of fatty acids and their correlation with calcium and fat absorption.

The goal of this study was to assess the comparative individual balance of fatty acids and associate their eventual interference with calcium absorption by normal term infants fed formulas containing distinct blends of fats: one with palm olein, palm kernel oil, and canola oil, and the other with a different oil composition.

## Methods

### Study design and subjects

**Fig. 1:**
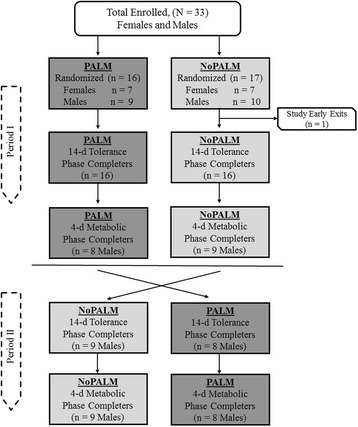
Study flow diagram of the progress through the periods and study subject dispositions [9]

This controlled, randomized, double-blinded, crossover balance study tested two formulas in normal infants (68–159 ± 3 days of age at enrollment) attending a daycare center in Salvador, Bahia, Brazil. Each feeding period had a 14-day tolerance phase and a 4-day metabolic balance phase (Fig. 1). The study was conducted according to the principles of the Declaration of Helsinki and Good Clinical Practices. The protocol was approved by the Institutional Research Board of the Federal University of Bahia, Salvador, Brazil. The parents and/or legal guardians of the infants provided written informed consent. The study was registered at clinicaltrial.gov (#NCT00941564) [9].

### Study feedings

The PALM formula, currently marketed under the name NAN PRO1™ (Nestle), includes 44.0% palm oil, 21.7% palm kernel oil, and 18.5% canola oil as the predominant fats and fish oil as a source of DHA. The protein source is whey:casein (70:30). The other formula (NoPALM; Similac Advance™), without olein palm or palm kernel oil, contained 41.4% high oleic sunflower oil, 29.6% coconut oil, and 27.6% soy oil as the major fats, algal oil (*Crypthecodinium cohnii*) as a source of DHA and *M. alpina* oil for ARA . The protein source is whey:casein (48:52). The two study formulas contained comparable levels of vitamin D and nutrient levels as recommended by the Brazil Ministry of Health [14] and Codex Alimentarius [15] (Table 1). The study investigators and the subjects’ parents were blinded to the dietary allocation. Data analysis was performed with the dietary groups coded, and the code was not broken until all of the analyses were completed [9].

**Table 1:** Approximate composition of formula products included in this study (per 100 g of powder)

Nutrient^*^	PALM	NoPALM	Brazil Human Milk Reference^a^
Energy, kcal	519	513	-
Protein, g	9.5	11	8.0
Carbohydrate, g	57.9	55	-
Fat, g	27.7	28	28.0
Palm olein Oil (%)	44	-	-
High Oleic Sunflower oil (%)	-	41.4	-
Palm Kernel Oil (%)	21.7	-	-
Coconut Oil (%)	-	29.6	-
Soy Oil (%)	-	27.6	-
Canola Oil	18.5	-	-
Corn Oil	10.9	-	-
Milk Fat	2.8	-	-
Others	2.1 ^b^	1.4 ^c^	-
Fatty Acids (g/100 g Fat) ^d^			
12:0	7.79	13.22	6.88 ± 2.79
14:0	3.17	5.02	7.02 ± 3.07
16:0	22.61	7.46	17.30 ± 2.2
18:0	3.42	3.11	5.30 ± 1.26
18:1n9	43.94	47.68	25.00 ± 3.46
18:2n-6	15.74	20.49	20.30 ± 6.48
18:3n-3	1.92	1.52	1.43 ± 0.66
20:0	0.41	0.21	0.12 ± 0.03
20:1n-9	0.35	0.24	0.26 + 0.06
20:4n-6	0.25	0.40	0.53 ± 0.14
22:0	0.22	0.49	Tr
22:6n-3	0.18	0.17	0.14 ± 0.05
Minerals			
Calcium, mg	279 ^d^	424 ^d^	246.50
Phosphorus, mg	160	216	122.60
Magnesium, mg	36	31	29.30
Vitamins			
D, μg	7.80	8.60	-

^*^ Values are Manufacturer’s Label Claims, except where stated [9]

^a^ Silva et al. [43]; Braga and Palhares [44]

^b^ DHA, ARA and Soy lecithin

^c^ DHA, and ARA

^d^ Investigator’s analytical for fatty acids

*Tr* trace

### Metabolic balance studies

Using a computer-generated randomization schedule, each infant was randomly assigned to receive one of the paired study formulas as their initial food source and fed for 14 days (tolerance study) before the start of the 4-day metabolic balance study (Period I). Following Period I, each infant received the other paired study formula for at least another 14 days prior to the second 4-day metabolic balance study (Period II) [9].

Male subjects who completed the first 14-day phase went through the additional 4-day metabolic phase assessment in both study periods. Female subjects did not undergo the metabolic assessment to avoid contamination of the stool samples with urine during sample collection. No breast milk supplementation was allowed during the metabolic study [9].

The metabolic studies with fecal and urine collections were performed over 4 days and three nights (72 h). The infants were kept on metabolic beds specially designed to accurately collect separate urine and stool samples [16]. To determine formula intake, the bottles were weighed before and after each feeding. Vomiting and spit-up losses were quantitatively assessed by pre-weighed bibs after feedings. The subjects’ weights were measured daily at the hospital by study staff members. The brilliant blue marker method was used to determine completion of intestinal transit of the test formulas [13, 17]. Daily fecal and urine samples were weighed, homogenized, and stored separately in containers at −80 °C for further analysis.

### Sample analyses

The total fat content of the formula and feces were extracted with chloroform:methanol (2:1) and quantified by gravimetry as described by Folch et al. [18]. The individual fatty acids were identified by gas chromatography. Aliquots of fat were saponified followed by methylation. Fatty acid methyl esters (FAME) were extracted and stored in an inert atmosphere (N_2_) in a freezer at −60 °C. Methyl tricosanoate 23:0 (T9900; Sigma Aldrich®), was added as an internal standard [19]. The FAME were separated and identified on a gas chromatograph (Varian® CP 3800) with a DB-FFAP column (30 m × 0.32 mm × 0.25 mm) and equipped with a flame ionization detector. The analysis parameters included an injector temperature of 250 °C and detector temperature of 280 °C. The following thermal program was used: 150 °C for 16 min, then increasing by 2 °C/min up to 180 °C, maintained for 25 min, following an increase of 5 °C/min up to 210 °C, maintained for 25 min. Helium was used as a carrier gas at 1.0 mL min^−1^. Nitrogen gas was used as the make-up gas (30 mL min^−1^); hydrogen gas and synthetic air flows were provided at 30 and 300 mL min^−1^, respectively. The injections were performed in duplicate for each extraction in a volume of 1 μL. FAME were identified by comparing retention times with known mix standards (189–19; Sigma-Aldrich®). The quantification of fatty acids, expressed in milligrams per gram of lipids, was performed by the addition of an internal standard (23:0). All analyses were conducted in duplicate. As a parameter for the conclusive identification of fatty acids, the samples were injected in a gas chromatograph coupled to a gas chromatography mass spectrophotometer (Clarus 500; Perkin Elmer®). The mass fragments of the samples were compared with the spectral data of the National Institute of Standards and Technology (NIST) standard mass spectral databases and mix standards under the same operating conditions used in the GC/FID at a spectrum of 50–500 m/z (EI, 70 eV).

The study formulas and fecal samples were also separately analyzed for calcium using an atomic absorption spectrophotometer (Model 55B; Varian Medical Systems) after acid digestion. Urine samples were directly analyzed using the atomic absorption spectrophotometer without acid treatment [9].

### Data and statistical analyses

Nutrient absorption was calculated as intake minus fecal excretion. Percent absorption was determined by dividing the amount absorbed by the intake amount, multiplied by 100. The primary study variable was calcium absorption calculated from calcium intake and fecal and urinary calcium. The secondary variables included calcium retention, fat absorption, and individual fatty acid absorption. Safety data included adverse events and serious adverse events.

Data were statistically analyzed using STATA 12 (StataCorp, College Station). The interaction between study formula and period (carryover effect) was used to analyze metabolic balance outcomes by analysis of variance (ANOVA). When the carryover effects were significant (*p* < 0.10), only Period I data results were valid. A nonparametric test was used (Wilcoxon Rank Sum) to test the hypothesis of the equality of the observed measurements, with the level of statistical significance set at *p* < 0.05. Partial correlations were determined by general linear models after adjusting for formula using IBM SPSS Statistics for Windows, version 20.0 (IBM Corp.). Analysis of covariance was utilized in evaluating nutrient (calcium, fat, individual fatty acid) absorption wherever there were significant differences in nutrient intake; using nutrient intake as a covariate. Sample size was estimated using standard deviation of the primary variable, calcium absorption from similar study populations. A sample size of 12 subjects (six per sequence A to B and B to A) had an 80% power to detect a difference of at least 15% in calcium absorption, assuming a standard deviation of 7.9%. Approximately eight subjects per sequence were enrolled to account for 25% attrition. In the tolerance phase, a sample size of 20 subjects per group had 80% power to detect a difference of at least 0.55 in mean rank stool consistency.

## Results

A total of 33 subjects were enrolled in the study, were randomly assigned to initial formula groups (PALM =16; NoPALM =17), and completed the first phase (14-day tolerance study) [9]. One subject receiving NoPALM was hospitalized for a serious adverse event (pneumonia) and exited the study prematurely. Thus, 32 subjects completed the tolerance phase; of these, 17 male infants were fed the assigned study formulas exclusively (no human milk) at the hospital ward (Period II, 4 days). There were no significant differences (*p* > 0.05) between the two feeding groups in study entry information, adverse events, and other demographic data (Table 2). The age of the study subjects ranged from 68 to 159 days (*p* > 0.05). The intake of formula (PALM and NoPALM) was similar between the groups, without significant differences (*p* > 0.05) (Table 3) [9].

**Table 2:** Baseline subject information^*^

	Variables							
	Gestational age (weeks)	*p*	Age at study entrance (days)	*P*	Birth weight (g)	*p*	Birth length (cm)	*p*
PALM (*n* = 16)	39.8 ± 1.2	0.481	117 ± 26	0.346	3333 ± 490	0.932	49.1 ± 3.7	0.965
NoPALM (*n* = 17)	39.5 ± 1.1		108 ± 27		3321 ± 330		49.1 ± 1.9	

^*^ Values are means ± SD [9]

**Table 3:** Intake and excretion of fat, fatty acids, and calcium of infants fed PALM and NoPALM formulas^*^

Variables	PALM (*n* = 17)	NoPALM (*n* = 17)
FORMULA INTAKE (g/kg/day)	111.69 (85.30–166.92)	115.88 (69.54–144.48)
FAT (g/kg/day)		
Intake	4.62 (3,51–7.88)	4.54 (2.75–5.70)
Excretion	0.22 (0.10–0.34)	0.14 (0.03–0.44)^a^
FATTY ACIDS (mg/kg/day)		
Lauric acid (12:0)		
Intake	414.68 (324.4–593.58)	600.36 (363.92–752.36)^b^
Excretion	4.08 (1.60–5.02)	1.74 (0.38–2.34)^e^
Myristic acid (14:0)		
Intake	159.46 (124.74–228.26)	227.80 (138.08–285.48)^b^
Excretion	3.00 (1.18–9.56)	2.80 (0.60–6.02)
Palmitic acid (16:0)		
Intake	1177.96 (921.50–1686.14)	320.14 (196.06–401.18)^b^
Excretion	29.42 (13.20–113.14)	12.28 (2.86–24.74)^b^
Stearic acid (18:0)		
Intake	172.14 (134.66–246.40)	133.22 (80.74–166.94)^b^
Excretion	5.40 (2.18–18.06)	7.48 (1.76–15.74)
Oleic acid (18:1n9)		
Intake	2471.8 (1932.86–3536.70)	2202.12 (1334.84–2759.58)
Excretion	21.86 (11.14–73.36)	21.14 (6.52–124.64)
Linoleic acid (18:2n6)		
Intake	825.86 (646.06–1182.14)	930.68 (564.14–1166.28)
Excretion	8.18 (4.38–20.90)	5.36 (0.92–10.42)^f^
Linolenic acid (18:3n3)		
Intake	96.66 (75.62–138.36)	68.88 (41.76–86.32)^b^
Excretion	0.58 (0.32–1.78)	0.18 (0.00–0.62)^g^
Arachidic acid (20:0)		
Intake	20.72 (16.20–29.64)	9.64 (5.84–12.08)^b^
Excretion	0.48 (0.18–1.48)	0.40 (0.00–0.88)
Eicosenoic acid (20:1n9)		
Intake	17.76 (13.90–25.44)	10.90 (6.60–13.66)^b^
Excretion	0.48 (0.20–1.04)	0.26 (0.00–0.98)^h^
Arachidonic acid (20:4n6)		
Intake	12.44 (9.74–17.82)	18.26 (11.08–22.88)^b^
Excretion	0.34 (0.00–0.68)	0.36 (0.00–2.24)
Behenic acid (22:0)		
Intake	21.72 (17.00–31.12)	44.72 (27.08–56.40)^b^
Excretion	0.56 (0.00–2.20)	1.64 (0.00–4.32)^b^
Docosahexaenoic acid (22:6n3)		
Intake	8.34 (7.00–12.80)	7.72 (4.68–9.68)^c^
Excretion	1.42 (0.00–7.42)	0.20 (0.00–2.72)^i^
Total Fatty Acids		
Intake	5388.26 (4215.20–7712.82)	4552.06 (2759.28–5704.42)^d^
Excretion	94.36 (52.34–251.62)	61.30 (13.82–78.38)^j^
CALCIUM (mg/kg/day)		
Intake	47.20 (35.90–70.30)	73.50 (44.60–92.20)^b^
Fecal Excretion	29.01 (9.06–49.99)	30.90 (3.00–50.53)
Urinary Excretion	1.62 (0.65–3.43)	1.53 (0.32–2.83)

^*^Values are median (min ± max). ^b^ = *p* < 0.001; ^c^ = *p* = 0.011; ^d^ = *p* = 0.005; ^h^ = 0.004; ^i^ = 0.011

^a^ = Significant carryover effect (*p* = 0.071); therefore, valid period I significant difference (*p* = 0.027)

^e^ = Significant carryover effect (*p* = 0.016); therefore, valid period I significant difference (*p* = 0.004)

^f^ = Significant carryover effect (*p* = 0.016); therefore, valid period I significant difference (*p* = 0.043)

^g^ = Significant carryover effect (*p* = 0.010); therefore, valid period I significant difference (*p* = 0.004)

^j^ = Significant carryover effect (*p* = 0.032); therefore, valid period I significant difference (*p* = 0.009)

A significant carryover effect in fecal excretion and absorption of fat was detected (*p* = 0.071 and *p* = 0.059, respectively); consequently, only Period I results were considered valid. In Period I, the stool fat content was significantly lower after NoPALM feeding than that after PALM feeding (*p* = 0.027). The NoPALM feeding group had significantly (*p* = 0.020) higher fat absorption (96.55%) than that of the PALM feeding group (95.50%) (Table 4) [9].

**Table 4:** Absorption of fat, fatty acids, and calcium of infants fed PALM and NoPALM formulas^*^

Variables	PALM (*n* = 17)	NoPALM (*n* = 17)
FAT		
Absorbed (g/kg/day)	4.38 (2.65–7.67)	4.39 (3.39–6.56)
Absorption (%)	95.50 (92.50–98.00)	96.55 (90.10–99.50)^a^
FATTY ACIDS		
Lauric acid (12:0)		
Absorbed (mg/kg/day)	412.70 (322.58–598.64)	598.72 (363.64–751.96)^b^
Absorption (%)	98.89 (97.44–99.54)	99.70 (99.57–99.95)^f^
Myristic acid (14:0)		
Absorbed (mg/kg/day)	157.34 (122.86–223.18)	224.42 (135.64–284.88)^b^
Absorption (%)	97.65 (93.90–98.75)	98.54 (98.23–99.79)^g^
Palmitic acid (16:0)		
Absorbed (mg/kg/day)	1154.38 (898.98–1620.56)	301.88 (183.54–398.32)^b^
Absorption (%)	97.56 (90.22–98.90)	95.77 (91.18–99.29)
Stearic acid (18:0)		
Absorbed (mg/kg/day)	167.98 (130.90–234.68)	125.10 (75.10–165.06)^b^
Absorption (%)	97.01 (89.32–98.75)	94.59 (86.51–98.88)
Oleic acid (18:1n9)		
Absorbed (mg/kg/day)	2449.10 (1915.80–3480.48)	2181.60 (1325.88–2753.04)^c^
Absorption (%)	98.89 (96.98–99.22)	98.91 (98.28–99.76)
Linoleic acid (18:2n6)		
Absorbed (mg/kg/day)	818.44 (642.22–1169.44)	926.72 (562.32–1165.36)
Absorption (%)	99.08 (97.42–99.37)	99.46 (98.77–99.92)
Linolenic acid (18:3n3)		
Absorbed (mg/kg/day)	96.02 (75.30–137.28)	68.76 (41.58–86.24)^b^
Absorption (%)	99.42 (98.13–99.60)	99.68 (99.02–100)
Arachidic acid (20:0)		
Absorbed (mg/kg/day)	20.34 (15.90–28.44)	9.22 (5.44–11.94)^b^
Absorption (%)	97.53 (92.70–99.11)	95.33 (89.66–100)^i^
Eicosenoic acid (20:1n9)		
Absorbed (mg/kg/day)	17.44 (13.58–24.60)	10.52 (6.60–13.60)^b^
Absorption (%)	97.44 (98.89–93.51)	97.68 (91.45–100)
Arachidonic acid (20:4n6)		
Absorbed (mg/kg/day)	12.16 (9.32–17.32)	17.96 (10.96–22.76)^b^
Absorption (%)	97.23 (93.86–97.64)	98.09 (96.47–99.46)^h^
Behenic acid (22:0)		
Absorbed (mg/kg/day)	10.74 (8.30–14.58)	21.56 (12.94–27.72)^b^
Absorption (%)	96.92 (92.88–100)	95.60 (88.94–100)
Docosahexaenoic acid (22:6n3)		
Absorbed (mg/kg/day)	7.28 (6.30–10.46)	7.32 (6.56–9.68)
Absorption (%)	95.23 (80.06–100.00)	97.44 (66.63–100.00)^l^
Total Fatty Acids		
Absorbed (mg/kg/day)	5325.34 (4162.64–7552.54)	4490.86 (2727.24–5690.6)^d^
Absorption (%)	97.92 (95.25–98.84)	98.54 (98.23–99.76)
CALCIUM		
Absorbed (mg/kg/day)	20.30 (0.10–37.10)	41.30 (25.20–92.20)^b^
Absorption (%)	40.90 (0.30–74,70)	58.00 (34.20–100.00)^e^
Retention (mg/kg/day)	17.90 (0.60–36.40)	39.60 (23.50–90.20)^b^
Retention (%)	38.70 (18.20–73.20)	55.10 (32.00–97.80)^m^

^*^Values are median (min ± max). ^b^ = *p* < 0.001; ^c^ = *p* = 0.037; ^d^ = 0.009; ^e^ = 0.015, using intake as a covariate *p* = 0.104; ^l^ = 0.038; ^m^ = 0.008

^a^ = Significant carryover effect (*p* = 0.005); therefore, valid period I significant difference (*p* = 0.020)

^f^ = Significant carryover effect (*p* = 0.005); therefore, valid period I significant difference (*p* = 0.001)

^g^ = Significant carryover effect (*p* = 0.028); therefore, valid period I significant difference (*p* = 0.005)

^h^ = Significant carryover effect (*p* = 0.040); therefore, valid period I significant difference (*p* = 0.021)

Intake, fecal excretion, and absorption of total fatty acids are summarized in Tables 3 and 4. The total fat intake was similar, but due to the different fatty acid concentrations (distinct sources of vegetable oils) of the evaluated formulas, there were significant differences in the intakes of individual and total fatty acids. When fed the NoPALM formula, the infants ingested significantly higher amount of lauric (12:0), myristic (14:0), arachidonic (20:4n6), and behenic (22:0) acids and significantly lower amount of palmitic (16:0), stearic (18:0), linolenic (18:3n3), arachidic (20:0), eicosenoic (20:1n9), and docosahexaenoic (22:6n3) acids compared to those fed the PALM formula. The intakes of oleic (18:1n9) and linoleic acid (18:2n6) were not significantly different between the two formulas. As expected, the intake of palmitic acid from the PALM formula (1177.96 mg/kg/day) was higher (about three times) than that of the NoPALM formula (320.14 mg/kg/day) (*p* < 0.05). The intakes of other fatty acids were relatively similar (Table 3).

The fecal excretion of total fatty acids was significantly (*p* < 0.05) higher in the PALM (94.36 mg/kg/day) than in the NoPALM (61.30 mg/kg/day) formulas. Palmitic and oleic acids were excreted at higher concentrations in the infants’ feces. The fecal excretions of palmitic and oleic acids were 38.96 and 28.94% in the PALM formula versus 23.16 and 39.87% in the NoPALM formula, respectively (Table 3).

The intakes and excretions of ARA and DHA differed between the evaluated formulas. For ARA, intake was significantly higher in the NoPALM formula (18.26 mg/kg/day) (*p* < 0.001) while excretion did not differ between formulas. However, the intake (8.34 mg/kg/day) and excretion (1.42 mg/kg/day) of DHA were greater for the PALM formula (*p* < 0.05) (Table 3).

The intestinal absorption of each individual fatty acid in the 17 infants is shown in Table 4. The absorption of total fatty acids from the PALM formula was less (97.92%) than that of the NoPALM formula (98.54%) (*p* > 0.05). The absorption of the saturated lauric (C12:0) and myristic C14:0 acids significantly better when the infants ingested the NoPALM formula. However, the intestinal absorption of palmitic (C16:0) and stearic (C18:0) acids was not significantly different between the formulas. It is worth noting that the absorption of the 18:2n6 (LA) and 18:3n3 (ALA) essential fatty acids (linoleic acid and α-linolenic acid, respectively) were similar in both groups, but the NoPALM feeding group had a statistically higher absorptions percentage of LCPUFA (ARA, *p* = 0.021; DHA, *p* = 0.038) than the PALM feeding group.

Calcium absorption, expressed as mg/kg/day or as percentage, was significantly higher after NoPALM than PALM ingestion (*p* < 0.001 and *p* = 0.015, respectively). Similar results were obtained for calcium retention, in which NoPALM feeding showed higher retention compared to that with PALM feeding (*p* < 0.001). However, when calcium intake was used as a covariate, the difference in calcium absorption was not significant (*p* = 0.104); however, calcium retention remained higher (*p* = 0.024) after ingestion of the NoPALM formula [9].

**Fig. 2:**
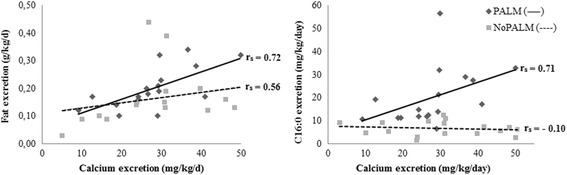
Correlations between fecal excretions of calcium, fat, and palmitic acid according to formula. r_s_ - Spearman correlation

Significant correlations were observed between fecal calcium excretion and fecal excretion of fat and the major fatty acids. The correlation between the fecal excretions of calcium and fat was significant and showed the highest correlation coefficient for the PALM formula (*r*_*s*_ = 0.72, *p* = 0.001) compared to that of the NoPALM formula (*r*_*s*_ = 0.56, *p* = 0.018) (Fig. 2). The fecal excretions of the major saturated and unsaturated fatty acids were significantly correlated with total calcium excretion for the PALM formula. The correlations were significant and positive for palmitic acid (*r*_*s*_ = 0.71, *p* < 0.000), stearic acid (*r*_*s*_ = 0.70, *p* = 0.002), oleic acid (*r*_*s*_ = 0.66, *p* = 0.003), and ARA (*r*_*s*_ = 0.57, *p* = 0.017). Fatty acids losses were directly and significantly correlated with fat excretion in the PALM formula. Palmitic acid was the major fecal fatty acid in most of the infants, and also showed the highest correlation coefficient (*r*_*s*_ = 0.85, *p* < 0.000), followed by stearic (*r*_*s*_ = 0.75, *p* < 0.000), oleic (*r*_*s*_ = 0.67, *p* = 0.003), and linoleic acids (*r*_*s*_ = 0.60, *p* = 0.011).

## Discussion

The results showed that, although formulas containing a mixture of palm olein, palm kernel oil, and canola oil (PALM) provide proportions of palmitic acid similar to those of human milk fat, they result in significantly lower absorption of fat and retention of calcium by infants compared with a blend of sunflower, coconut, and soy oils (NoPALM) (Tables 1 and 4). Furthermore, we observed different absorption percentages for fatty acids in the formulas examined in this study (Table 4). The results are concordant with those of other studies [4, 9, 12, 13] comparing calcium and fat absorption from palm olein–predominant milk-based formulas versus formulas without palm olein.

Compared to calcium absorption, calcium retention is more accurate markers of functional outcomes for the impact of dietary calcium on calcium homeostasis [9, 20]. Borschel et al. [21] demonstrated a significantly (*p* = 0.041) lower bone mineral content in term infants fed a palm olein containing partially hydrolyzed whey protein-based formula compared to a similar formula containing no palm olein. In another clinical trial, quantitative balance studies were performed to compare calcium absorption in healthy, full term infants fed casein hydrolysate-based and soy protein-based infant formulas with or without palm olein.

In another clinical trial, quantitative balance studies were performed to compare calcium absorption in healthy, full term infants fed casein hydrolysate-based and soy protein-based infant formulas with or without palm olein. Calcium intake did not differ between the groups. However, infant’s calcium absorption was less when fed with casein hydrolysate-based and soy protein-based with palm olein compared to when fed without olein [13].

It is important to highlight that the fat absorptions for both formulas assessed in this study (PALM, 95.50%; NoPALM, 96.55%) were comparable to that of human milk (90.5–97.10%) [5, 22]. However, only the NoPALM formula offered calcium absorption and retention (58.00 and 55.10%, respectively) similar to the reported values of breast milk (58.70 and 52.40%, respectively) [23]. An important physiological consequence of reduced calcium bioavailability is the negative effect on bone mass accretion. Moreover, good fat absorption is important for infants because of the high calorie content of fat and its role in brain development [24].

A systematic review of human intervention studies on the effects of infant formulas with the addition of palm olein on bone mineral content and bone mineral density concluded that healthy infants fed a formula containing palm olein as the predominant oil had significantly lower values for both parameters than those fed a formula without olein. The inclusion of this oil in infant formula to provide a fatty acid profile at required levels leads to lower bone mineralization [6].

Infants fed NoPALM in the current study had significantly higher fecal concentrations of oleic, palmitic, and stearic acids versus higher fecal concentrations of palmitic, oleic, and linoleic acid after PALM formula feeding. Palmitic acid accounted for a large proportion of unabsorbed fatty acids in the PALM formula (38.96%). Studies have reported that infants fed a milk-based formula containing palm olein as a predominant fat have a higher fecal excretion and lower absorption of palmitic acid [4, 11, 12]. In palm olein, the palmitic acid is preferentially esterified at positions sn-1 and sn-3 of the triglyceride molecule. Thus, it is absorbed as a free fatty acid that can bind with calcium in the intestine, forming fatty acid soaps that are excreted fecally, resulting in the low absorption of both nutrients. Moreover, in the intestine, the fatty acid soaps solidify because of their high melting temperature causing hard stools and constipation in the infant. [6, 8, 9, 13].

As previously demonstrated [4, 12], the percentage of palmitic acid absorption was similar for the PALM (97.56%) and NoPALM (95.77%) (*p* = 0.094) formulas (Table 4). Nelson et al. [4] compared the absorption of fatty acids in a group of term infants fed a milk-based formula containing palm olein (45%), soy (20%), coconut (20%), and sunflower (15%) oils with a group fed a formula that contained a blend of safflower, coconut, and soy oils, concluding that the absorption of palmitic acid (91.70%) was better in the formula without palm olein. However, unlike the study of Nelson et al. [4], the PALM formula in the current investigation contained palm kernel oil in addition to palm olein. While palm olein is extracted from the mesocarp of the fruit *Elaeis guineans*, the palm kernel oil is derived from the seed of this fruit and the two have different fatty acid compositions. Palm olein contains 40–42.5% palmitic acid, 9% of which is esterified in the sn-2 position, 9.4–13.52% is already in kernel oil, and 6% is in the sn-2 position [22, 25]. This change in fat composition may influence the fatty acid absorption by infants.

The lauric (C12:0) and myristic (C14:0) saturated fatty acids were significantly (*p* < 0.05) better absorbed by infants fed the NoPALM (99.70 and 98.54%, respectively) formula compared to those fed the PALM (98.89 and 97.65%, respectively) formula. Raiten [1], as in the Assessment of Nutrient Requirements for Infant Formulas report, did not recommend adding myristic or lauric acids to infant formulas since there are no data to indicate their specific roles as dietary nutrients. However, these fatty acids are components of some oils used in infant formulas, and the author does not proscribe the use of such oils [1]. Since no data are available on which to base a recommendation, the Codex Alimentarius [15] recommends that the maximum levels of lauric and myristic acid in infant formulas not exceed 20% of the total fatty acids. The evaluated formulas had concentrations within these values (PALM, 12.26%; NoPALM, 18.99%) (Table 1). Furthermore, the infants fed both formulas demonstrated absorption percentages of lauric and myristic acid similar to those of infants fed breast milk [5].

The absorption of essential fatty acids (18:2n6 and 18:3n3) were similar for both formulas. However, the absorption of LCPUFA (ARA and DHA) were significantly (*p* < 0.05) greater for the NoPALM formula even when the intake was used as a covariate (Tables 3 and 4). The values found in this study were superior to those found by Moya et al. [26] and Canielli et al. [27]. However, both of those studies measured the absorption of fatty acids in premature infants, which may explain the lower values. To date, our current study is the first and only study to report the impact of dietary palm olein on the absorption of DHA and ARA in infants. Previous studies on palm olein evaluated infant formulas which were not supplemented with DHA and ARA.

The importance of essential fatty acids, as dietary precursors for eicosanoid and docosanoid formation, has been widely reported. The LCPUFA DHA and ARA are derived from their precursors ALA and LA, respectively. However, ALA and LA cannot be synthesized owing to the lack of the required dietary enzymatic desaturases [28]. DHA and ARA are found in high proportions in the structural lipids of cell membranes, particularly those of the retina and central nervous system, and their accretion primarily occurs during the last trimester of pregnancy and the first year of life [28, 29].

It had previously been assumed that infants could synthesize LCPUFA from essential fatty acids (ALA and LA) through the elongase and desaturase systems. However, evidence that infants fed formula deficient in LCPUFA have a significantly lower plasma or red blood cell levels of DHA and ARA compared with those who were breastfed or fed formula supplemented with LCPUFA suggests that the enzyme systems in infants may be inefficient during the first months of life [30].

In the neonatal period, dietary n-6 and n-3 fatty acid balance is necessary to provide essential polyunsaturated fatty acids for normal growth and development, particularly that of the brain. This ratio is important because both essential fatty acids (ALA and LA) compete for the same enzyme during the synthesis of LCPUFA (DHA and ARA). In this study, both formulas were within the margin of 5:1 and 15:1 suggested by the Codex Alimentarius [15] (PALM, 8:1; NoPALM, 12:1) and the ratio reported in breast milk (10:1) [5, 27].

Supplementation of infant formulas with DHA and ARA for term infants remains controversial. A meta-analysis by Qawasmi et al. [31] concluded that the supplementation of infant formulas with LCPUFA failed to show any significant effect on improving early infant cognition; however, opposite results were reported by Jiao et al. [32]. Another meta-analysis showed that LCPUFA supplementation of infant formulas improves infant visual acuity up to 12 months of age [33]. The European Food Safety Authority concluded from a review of the literature that, although DHA is required for infant formula, ARA is not [34]. However, Crawford et al. [35] did not agree with this opinion and have commented on the recommendations around the need for ARA in infant formulas.

The data presented in this study show that the absorptions of the fatty acids DHA and ARA were as efficient as those from breast milk [27] for the two evaluated formulas. However, absorption percentages were significantly higher for the NoPALM formula.

Fish and algal oils are the main sources of DHA added to infant formulas. However, unlike in breast milk triacylglycerols, in which DHA is preferentially esterified in the sn-2 position, algal and fish oils do not have a strong positional specificity; rather, there are similar proportions at the sn-1, sn-2, and sn-3 positions [36]. Differences in the molecular structure of the triacylglycerols in these oils may contribute to the differences in digestibility and absorption of these two products [37].

Our results showed that DHA was better absorbed by infants fed the NoPALM formula than those fed the PALM formula. The source of DHA may partly explain this difference (NoPALM, algal oil; PALM, fish oil) since the intake of this fatty acid was significantly higher with the PALM formula. Clandinin et al. [38] evaluated the benefits of feeding preterm infants formula supplemented with fish and algal oils as a source of DHA. The authors observed an increase in weight and length of infants fed DHA from algal oil but not from fish oil; however, the mechanism for this increase was unclear. Tou et al. [39] also observed the influence of DHA source in digestibility and tissue incorporation of rats fed diets containing different oils. Unlike the above studies in preterm infants and rats, the source of DHA is less likely to be impactful on DHA absorption in comparison to the impact of palm olein in our current study because we evaluated term human infants and there were no differences noted in weight or growth. Nonetheless, the influence of DHA sources on DHA absorption human term infants remains untested.

An association between the fecal excretions of calcium and fatty acids, especially palmitic and stearic acids, was demonstrated for the PALM formula. The increase in calcium excretion was significantly (*p* < 0.01) and directly proportional to the excretions of palmitic and stearic acid in the PALM formula (*r*_*s*_ = 0.71 and *r*_*s*_ = 0.69, respectively). However, these correlations were inversely proportional but not significant (*p* > 0.05) in the NoPALM formula (*r*_*s*_ = −0.10 and *r*_*s*_ = −0.40, respectively) (Fig. 2). These data reinforce the hypothesis that the excretion and consequent absorption of calcium are closely related to the palmitic acid source in infant formula. The palmitic acid from palm olein is not absorbed efficiently; rather, it forms insoluble calcium soaps in the intestinal tract, rendering a portion of dietary calcium unavailable for absorption. The observation of a high correlation between calcium and palmitic acid excretion in infants fed formula containing palm olein resulting in low calcium absorption and retention is also supported by other authors [4, 12].

Whether the reduced absorption percentage of fat, fatty acids, and calcium retention caused by the inclusion of palm olein in infant formula is clinically relevant is a matter of perspective. Fecal loss of 0.22 g fat/kg (PALM) and 0.14 g/fat/kg (NoPALM) represents a loss of 9.95 kJ/kg (2.4 kcal) and 6.30 kJ/kg (1.50 kcal), respectively, each day. Normal infants are certainly capable of increasing energy intake proportionately to make up for an energy loss of this magnitude, but preterm infants may have difficulty because of intestinal immaturity. The fecal loss per day can be considered low, but should be taken into consideration during the first year of life when infants are almost exclusively fed formula. As fat provides up to 50% of the total calorie content of most infant formulas, it is important to make allowance for the variations in absorption with different sources of fats. Estimation of caloric intake on the basis of milk composition alone is likely to be a confounding factor when fats of different origins are considered. Similarly, decreased retention of calcium suggests decreased bone mineral deposition. Koo et al. [40] demonstrated that differences in calcium absorption in infants fed formulas with and without palm olein, led to significant differences in bone mineral content at three and 6 months of age. However, additional long-term studies are necessary to evaluate this influence.

A limitation of our current concerns the carryover effect, observed for some variables, between the analyzed periods, wihich can distort the results obtained after the second period. The crossover design is used in clinical trials to provide an unbiased estimate of the difference between the treatment effects. In the presence of a differential carryover effect, such an estimate can only be obtained by: using data from the first treatment period only or assuming that there is no differential carryover [41]. According to William and Pater [42] in many situations the carryover effect is unlikely to exist. However, these authors and others advise that if crossover design has been used, unless carryover effects are negligible, the analysis is based on only the first-period data. But, evaluating only the data of the first period, is a limiting factor, because it increases the variance by not eliminating the variability between subjects. In other studies, the use of washout periods between administrations of interventions can be used to combat carryover effects.

## Conclusions

The use of powdered formula that is free of palm olein and palm kernel oil is associated with improved intestinal absorption of the major fatty acids and total fat, as well as calcium retention. The infants showed a similar absorption of essential fatty acids LA and ALA; however, the NoPALM-fed infants had higher absorptions percentage of LCPUFA (ARA and DHA). Moreover, the DHA source in the NoPALM formula (algal oil) was better absorbed than the source in the PALM formula (fish oil). Our study illustrates the important general point that differences in formula composition may have a significant effect on the physiologic response of infants. This emphasizes the need for research on the efficacy and safety of new formulations of breast milk substitutes even when they conform to international guidelines for overall nutrient composition. It is important to stress that the findings of our study of Brazilian infants were consistent with those of previous studies on palm olein–predominant formula, despite differences in other fats.

## Abbreviations

ALA: α-linolenic acid
ARA: arachidonic acid
DHA: docosahexaenoic acid
FAME: fatty acid methyl esters
LA: linoleic acid
LCPUFA: long-chain polyunsaturated fatty acids
NoPALM: milk-based powdered commercial formula containing no palm olein
PALM: milk-based powdered commercial formula containing palm olein and palm kernel and canola oils as the predominant fats
